# Acute insomnia as the initial manifestation of Wilson’ s disease: A Case Report

**DOI:** 10.1097/MD.0000000000039380

**Published:** 2024-08-16

**Authors:** Xiaofang Cheng, Quanhui Chen, Haoyuan Ma, Qiuxia Ren, Shusheng Jiao

**Affiliations:** a Department of Neurology, Bethune International Peace Hospital, Shijiazhuang, Hebei, China; b Department of Gastroenterology, Bethune International Peace Hospital, Shijiazhuang, Hebei, China.

**Keywords:** insomnia, MRI, sleep disorders, Wilson’s disease.

## Abstract

**Background:**

Wilson’s disease (WD) is a rare autosomal recessive disease that causes impaired copper circulation and excretion. The initial manifestations of WD vary clinically, which makes early diagnosis very difficult. Sleep disorders have been described as common symptoms of WD, but the initial manifestations are in rare cases.

**Case report:**

This study aims to present a patient with acute insomnia as the initial manifestation of WD. Cranial magnetic resonance imaging showed extensive lesions in the bilateral putamen and caudate nucleus, pressure area of corpus callosum, midbrain, and pons. Interestingly, rare but characteristic signs of WD, such as “face of the giant panda,” were shown in this case. WD diagnosis was further established by decreased ceruloplasmin level and *ATP7B* (adenosine-triphosphatase copper transporting beta polypeptide) gene mutations.

**Conclusions:**

We describe acute insomnia as the initial manifestation of WD in a 21-year-old male patient. Timely diagnosis allows for early copper-eliminating pharmacotherapy, which is of high prognostic importance, as the patient may be more responsive to treatment at this point.

## 1. Introduction

Wilson disease (WD), also known as hepatolenticular degeneration, is an autosomal recessive disease with an incidence of < 3/100,000, which is a rare genetic disease. WD is caused by mutations in the *ATP7B* (Adenosine-Triphosphatase Copper Transporting Beta Polypeptide) gene on chromosome 13. The *ATP7B* protein plays an important role in copper homeostasis.^[1]^ *ATP7B* mutations lead to reduced copper binding by ceruloplasmin with free copper accumulating mainly in the liver, brain, cornea, kidney, and other organs. Symptoms of copper accumulations range from neurological and psychiatric disturbances to acute or chronic liver disease. Patients with WD have great variability in the symptoms, which makes early diagnosis difficult. The average onset age ranges between 5 and 35 years, and the prognosis tends to be worse if the diagnosis is delayed.^[2]^ Delays in diagnosis are common, meaning that opportunities to initiate chelation therapy and prevent neurological dysfunction were missed. In Ireland, the mean interval in patients diagnosed between 1990 and 2011 was 21 months and had not improved over the preceding 4 decades.^[3]^ The longest delays are often in patients who have psychiatric symptoms as initial presentation.^[4]^ Delays usually arise because the clinical features associated with neurological presentation are highly variable and initially misattributed to more prevalent disorders.^[5]^ Importantly, WD is potentially treatable if the treatment is started early.

Most patients with WD develop hepatic presentation (50%–60% cases) as the initial symptoms, ranging from clinically asymptomatic elevations in liver enzymes, through to hepatosplenomegaly, fatty liver disease, acute hepatitis, cirrhosis, and in rare cases, to acute liver failure.^[6]^ Regardless of the severity of any underlying liver disease, up to 60% of patients have neurological or psychiatric symptoms at onset, including dystonia, ataxia, tremor, choreoathetosis and parkinsonian-like extrapyramidal signs, depression, anxiety, and sleep disorders (SDs).^[1,7]^ Psychiatric symptoms can be the first manifestation and lead to diagnosis in almost 25% of patients with WD.^[8]^ Interestingly, a variety of SDs including insomnia, daytime sleepiness, poor sleep quality, rapid eye movement sleep behavior disorder (RBD), cataplexy-like episodes, and sleep paralysis have been described as common symptoms of WD.^[9,10]^ Studies report an incidence of SDs in WD of between 42% and 80%, a systematic review and meta-analysis in 2020 indicated 54.1% of patients with WD reported SDs, presenting a 7.7-fold higher odds of SDs compared to control patients.^[11]^ Besides, it has been shown that patients with WD with isolated liver disease and normal magnetic resonance imaging (MRI) features of the brain may also have SDs.^[11]^ Jernajczyk et al^[12]^ compared the video-polysomnography of WD patients with controls and found that WD patients had shorter mean total sleeping time, longer sleep latency, and lower sleep efficiency, and SDs tended to be worse in patients with neurological WD compared with hepatic WD. Although SDs are common manifestations of patients with WD, diagnosis is difficult to establish when patients develop SDs as the initial manifestations and available data are limited at present. Most studies on SD included patients with longstanding WD, in whom SDs appear later than liver and neurological symptoms, only several studies report RBD as the earliest symptom of WD.^[12–14]^

## 2. Case presentation

A 21-year-old male patient, who gave his written informed consent for publication, was admitted to the Neurology Department of our hospital in 2023 due to insomnia for 2 months, dysphagia and dysarthria for 1 month. He was a second-year undergraduate student and was healthy in the past, denied a history of insomnia and genetic disease in his family. Two months ago, the patient had symptoms of insomnia suddenly, which manifested as having trouble falling asleep, and his daytime symptoms were fatigue and memory problems, which made him feel distressed. He visited the outpatient clinic in other hospitals, was diagnosed with insomnia, and received non-benzodiazepines for anti-insomnia treatment. Unfortunately, the treatment efficacy was not good, the patient’s insomnia symptoms had not been significantly improved, which affected his grades.

**Figure 1.**
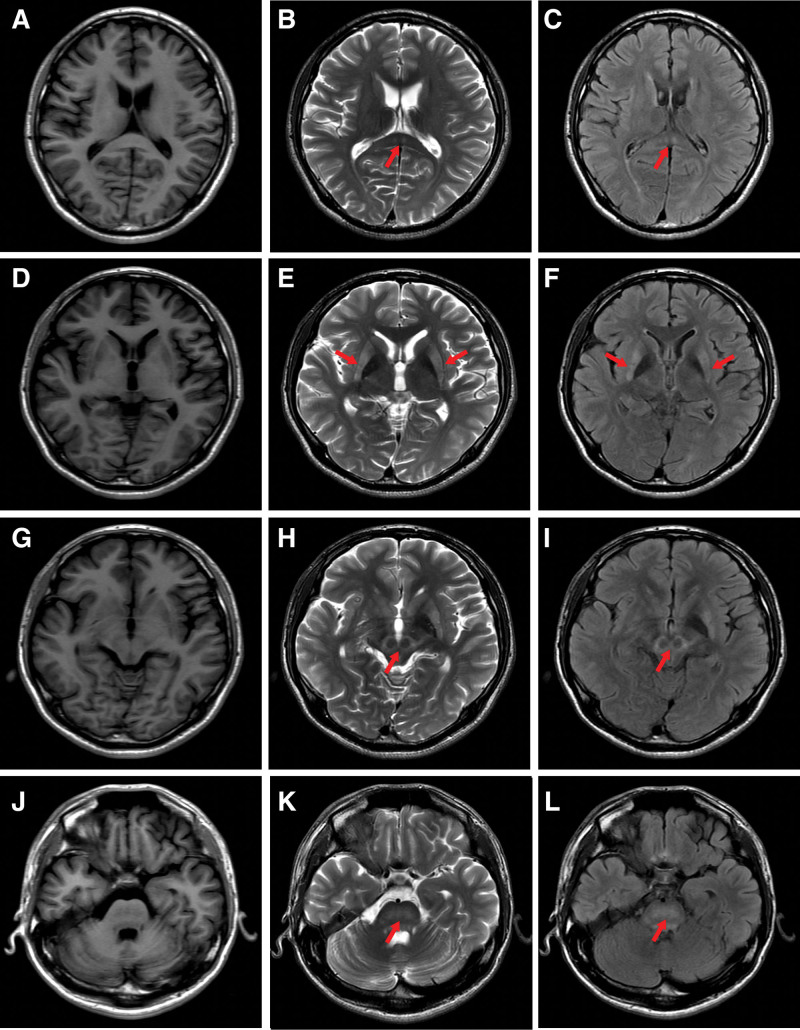
Representative magnetic resonance images of this patient. (A–C) T2-weighted and fluid-attenuated inversion recovery (FLAIR) axial magnetic resonance imaging (MRI) demonstrates hyperintensities in pressure area of corpus callosum (red arrow). (D–F) T2-weighted and FLAIR axial MRI demonstrates hyperintensities in bilateral caudate nucleus and putamen (red arrow). (G–I) T2-weighted and FLAIR axial MRI demonstrates hyperintensities in midbrain, and“face of the giant panda” (red arrow). (J–L) T2-weighted and FLAIR axial MRI demonstrates hyperintensities in pons, and central pontine myelinolysis–like signal changes (red arrow).

A month ago, the patient gradually developed symptoms of dysphagia and dysarthria, as well as decreased energy and appetite, and felt depressed. To further clarify the disease, the patient went to the outpatient clinic of our hospital and was admitted to the Neurology Department. After admission, the physical examination showed mild limb ataxia and bradykinesia, which were not noticed by the patient himself. The score of the Pittsburgh Sleep Quality Index was 12 points. The Cranial MRI showed extensive T2-weighted imaging (T2WI)/fluid-attenuated inversion recovery (FLAIR) hyperintense lesions in bilateral putamen and caudate nucleus, pressure area of CC, midbrain, and pons. Interestingly, MRI disclosed infrequent but characteristic lesions of WD, including “face of the giant panda” and central pontine myelinolysis (CPM)-like signal changes (Fig. 1). WD diagnosis was established with low ceruloplasmin level (0.03 mg/dL), high copper content in daily urine collection (145 μg/24 hour), and the presence of Kayser-Fleischer rings in the cornea. Genetic testing further confirmed the diagnosis, which detected heterozygosity for the c.-119_-118insCGCCG and c.-75C > A mutations of the *ATP7B* gene, both of which are classified as pathogenic. The patient received a score of 7 on the Leipzig scoring system for WD. Complete blood counts were normal. Liver function results were as follows: serum total protein of 68.0 g/L, albumin of 44.9 g/L, total bilirubin level of 17.8 μmol/L, alanine aminotransferase (ALT) level of 24.5 IU/liter, alkaline phosphatase (ALP) level of 110.0 IU/liter. Abdominal CT showed increased density of liver parenchyma, with a CT value of approximately 70-80 HU, as well as a widened portal vein.

After diagnosis, the patient was strongly advised to avoid copper-rich foods and beverages and received sodium dimercaptosulphonate 750 mg/d for treatment, as well as antidepressant and anti-insomnia treatments. After 2 courses of dimercaptosulphonate, an oral medication regimen was given. The therapy regimen was D-penicillamine 750 mg/d, vitamin B_6_ 50 mg/d, escitalopram oxalate 10 mg/d, and zolpidem 10 mg at night. When the patient was followed up 2 months after treatment, he reported a slight improvement in neurological symptoms and insomnia symptoms.

## 3. Discussion

We describe a detailed case of acute insomnia as the initial symptom of WD and was WD-related SDs, which adds a new insight to the notorious diverse spectrum of possible initial presentations of WD. Data on sleep in WD are scarce. In 2016, Tribl et al compared 41 patients with WD to healthy controls and found that WD showed significantly worse sleep quality, less sleep efficiency, increased wakefulness after sleep onset, and more arousal. Of these 41 patients with WD, 19 patients complained about current and prior insomnia, 3 patients reported insomnia as a first symptom of WD, which was determined retrospectively and lacked detailed case reports. As for SDs as the first symptoms of WD, most studies focused on RBD and demonstrated that RBD can appear as a prodromal phase phenomenon in WD.^[12,13]^

The high prevalence of SDs may be related to pathologic accumulation of copper in sleep/wake pathways in the brain,^[15]^ WD treatment,^[16]^ and other WD-related symptoms (i.e., motor, dysautonomic, psychopathologic, and metabolic disorders).^[1]^ Neuronal lesions in the brain of WD patients involve most of the sleep-wake regulation pathways, including brainstem nuclei, dentate nucleus, pons, thalamus, basal ganglia, external capsule, claustrum, and frontal lobes. Besides, pathological copper accumulation in the brain, especially in the brainstem, affected monoaminergic neurons (so-called rapid eye movement-off neurons) and impaired γ-amino butyric acid-mediated neurotransmission, which were vital in sleep/wake pathways.^[17,18]^ In this case, cranial MRI revealed diffuse lesions in the brain, including midbrain, pons, and basal ganglia, which were involved in the sleep/wake pathways. As the patient developed insomnia before any other symptoms and any treatments, acute insomnia may be the manifestation of the impairment of these sleep/wake pathways.

Interestingly, besides common lesions in WD, such as basal ganglia and brainstem lesions, this patient also had unusual but characteristic features of WD, such as “‘face of giant panda’” sign and CPM-like signal changes.^[19]^ These features help us diagnose this patient as WD at an early stage after admission. Brain lesions observed in WD are usually bilateral and symmetrical. Putamen and globus pallidus are particularly affected, but the caudate nucleus, internal capsule, substantia nigra, thalamus, cerebral cortex, subcortical white matter, subthalamic nucleus, cerebellum, and brainstem are also observed to have pathological changes.^[2]^ Brain MRI is a particularly important and useful tool for the diagnosis of WD and has demonstrated a significant correlation with clinical findings.^[20]^ Patients with neuropsychiatric form of WD invariably have abnormal MRI^[21]^ and even asymptomatic patients have been found with changes.^[22]^ These abnormalities are diverse, dynamic, and involve most of the structures of the brain in varying combinations and are classically localized in globus pallidus, putamen, thalamus, mesencephalon, pons, and dentate nucleus. In 90% of patients with neurological symptoms, hyperintense signal abnormality in the basal ganglia, thalamus, and brainstem on T2WI/FLAIR sequences is seen.^[23]^ The most frequent finding is the hyperintensity of T2WI in the basal ganglia.^[6]^

In recent years, some unusual MRI findings have been reported, such as “face of the giant panda,”^[24,25]^ lesions of CC,^[26]^ CPM-like signal changes,^[19]^ and so on. “‘Face of giant panda’” sign, where hyperintense signal abnormality surrounds the red nucleus and substantia nigra, is infrequent but more widely known in patients with WD,^[27,28]^ it provides a definite clue to the diagnosis of WD. Besides, lesions of CC have not been studied in a broad population of patients with WD,^[26]^ and WD patients with CC involvement have been found with more extensive brain lesions, more severe neurological dysfunctions and psychiatric symptoms.^[26]^ In this patient, the CC involvement was obvious on T2WI/FLAIR sequences and the lesions in brain were extensive, which was consistent with the abovementioned study. There are only a few reports of CPM-like changes in WD, and those with CPM-like changes have significantly more drooling and pseudobulbar features.^[29]^

To ascertain the value of various MRI features in differentiating neuropsychiatric form of WD from other early onset extrapyramidal disorders, Prashanth et al conducted a retrospective analysis included 100 patients, in which the following MRI observations were noted exclusively in WD: “‘face of giant panda’” sign (14.3%), tectal plate hyperintensity (75%), CPM-like abnormalities (62.5%), and concurrent signal changes in basal ganglia, thalamus, and brainstem (55.3%).^[19]^ Almost all of these exclusively MRI features were observed in this patient, except for thalamic lesions. Although cranial MRI features are important in the diagnosis of WD, the correlation between T2WI hyperintensities and clinical severity has not yet been described. Some semiquantitative MRI scales for measuring neuroradiological abnormalities in WD and quantitative MRI analyses have been carried out.^[5]^ Further studies exploring how imaging biomarkers correlate with clinical and biochemical characteristics are needed.

In WD, highly effective copper-eliminating pharmacotherapy is available and should be applied as soon as any symptom appears, which allows slowing down or almost stopping the cascade of brain tissue destruction and is beneficial for the long-term outcome in WD. Most encouragingly, the disappearance of numerous WD lesions was documented in an MRI study before and after D-penicillamine treatment with a follow-up period of 5 to 24 months, especially in the thalamus and the brainstem.^[30]^ The midbrain and tegmental pons have been shown to be the brain structures with the highest MRI signal normalization under WD-specific treatment.^[31]^ This suggests that early and correct diagnosis of WD is critical, which may delay or even reverse the course of the disease. The delay in diagnosis of WD may be attributed to the age of presentation and the primary features vary widely. Studies present that SDs are frequent but underdiagnosed in WD patients, and SDs may anticipate more severe psychiatric disorders.^[32]^ affecting quality of life, and compliance with WD treatment.^[33,34]^ Hence, for young patients with acute insomnia or SDs as initial symptoms, we should consider the possibility of WD, as early diagnosis may help to avoid further serious psychiatric and neurological complications. Awareness of the possibility of insomnia as a presenting clinical symptom of WD allows for early diagnosis of special cases, which is of high prognostic importance.

## 4. Conclusions

We report a young male WD patient with acute insomnia as the initial symptom, which is a detailed case report. This patient had rare but characteristic features of WD, such as “‘face of giant panda’” sign and central pontine myelinolysis (CPM)-like signal changes on cranial MRI. These findings have great significance for the study of the imaging characteristics of WD. The interval from onset to diagnosis of WD in this case was 2 months, timely and effective copper-eliminating pharmacotherapy was given to the patient, which may slow down the cascade of brain tissue destruction and is beneficial for the long-term outcome of WD. This case report has important suggestive significance for clinicians to diagnose WD at an early stage.

## Acknowledgments

The authors extend their gratitude to this patient for his cooperation in this study.

## Author contributions

**Conceptualization:** Xiaofang Cheng

**Data curation:** Xiaofang Cheng

**Methodology:** Xiaofang Cheng, Shusheng Jiao

**Writing – original draft:** Xiaofang Cheng, Shusheng Jiao, Qiuxia Ren

**Writing – review and editing:** Xiaofang Cheng, Quanhui Chen, Haoyuan Ma

**Investigation:** Quanhui Chen
